# A qualitative study on the willingness and influencing factors of master of geriatric nursing specialist postgraduates to volunteer for home respite care for disabled elderly families

**DOI:** 10.1186/s12912-024-01710-9

**Published:** 2024-01-17

**Authors:** Bihui Chen, Haili Zhu, Han Fu, Qiannan Han, Lei Chen

**Affiliations:** 1 College of Nursing, Hunan University of Chinese Medicine, No.300, Xueshi Road, Hanpu Science and Education Park, Yuelu District, Changsha City, Hunan Province China; 2 Nursing Department of Hunan Provincial Hospital of Integrated Traditional Chinese and Western Medicine, 58 Lushan Road, Changsha City, Hunan Province China; 3 Changsha Hospital of Traditional Chinese Medicine, No. 22, Xingsha Avenue, Changsha County, Changsha City, Hunan Province China

**Keywords:** Geriatric nursing, Postgraduate, Respite care, Volunteer service, Qualitative research

## Abstract

**Background:**

As China’s population ages, the demand for care for the disabled elderly is increasing, and family caregivers find it challenging to meet the comprehensive care needs of the disabled elderly. Through home respite services, families of the disabled elderly can receive help and support from specialized nursing professionals to ease the burden on family caregivers and provide high-quality services. This study explores the willingness and influencing factors of Master of Geriatric Nursing Specialist postgraduates in China to volunteer to provide home respite services for disabled elderly individuals.

**Methods:**

A qualitative study based on Grounded Theory used Strauss and Corbin’s programmatic version. A purposive sampling method was employed to conduct semi-structured interviews with 12 Master of Geriatric Nursing Specialist postgraduates from a tertiary hospital in Changsha, Hunan Province, China.

**Results:**

The willingness of Master of Geriatric Nursing Specialist postgraduates to volunteer to provide home respite services for the disabled elderly was established as a core category, which was influenced by three main categories: personal factors, service object factors, and social factors, and nine categories formed from 39 initial concepts were included under the main category.

**Conclusions:**

Influenced by China’s traditional cultural background, Master of Geriatric Nursing Specialist postgraduates in China have shown high motivation in volunteering to provide home respite services for the families of the disabled elderly but have been challenged by several challenges from China’s healthcare environment and education system. Relevant departments need to adopt a series of policies and measures to increase volunteers’ willingness to participate in respite care and promote its development.

## Background

The origins of respite services can be traced back to the 1940 and 1950 s in the United Kingdom [1, 2]. However, the deinstitutionalization movement of the 1960 and 1970 s in some European and American countries marked a significant turning point in the movement for reform of care practices [3, 4]. As the movement towards deinstitutionalization gained momentum, individuals with disabilities and chronic conditions were increasingly being cared for at home. This new situation has brought their problems to the fore, and the concept of respite care has emerged as a means of helping these families. There is no unified definition of respite care in the academic circle [5], and the specific definition of respite care may vary according to the needs of the recipients and caregivers. The definition given by the ARCH National Respite Network and Resource Center is considered to be generally applicable: Respite care is planned or emergency services that provide a caregiver of a child or adult with a special need some time away from caregiver responsibilities for that child or adult, and which result in some measurable improvement in the well-being of the caregiver, care receiver, and/or family system [6]. The Home and Community Based Service (HCBS) Waiver program [7] was established in 1981 in the United States, and the Home and Community Care Program(HACC) was established in 1985 in Australia [8] as a policy for early respite care. With the development of respite care, the services have covered families with notable groups such as the elderly, the young, the sick, and the disabled [9–11]. The mode and content of respite care have also become flexible and diversified, showing personalized development. There is a division between in-home respite and out-of-home respite based on the location of the service [12, 13], with in-home respite being the preferred mode of service for most families. However, a Norwegian study showed significantly higher rates of use of out-of-home respite care services for men with dementia and men living in urban areas [14]. Divided into planned and emergency respite care based on carers’ use [15, 16], a UK study has found that access to emergency respite and support may help stop people with a learning disability from needing to go to hospital for their mental health [17]. According to the professionalism and organization of service providers, they are divided into formal respite care and informal respite care [18, 19]. A study in the United States found that using a mixture of formal and informal respite care had a higher positive effect on family stability than either mode alone, while parents who received only informal respite reported lower benefits [18]. In addition, during the COVID-19 pandemic, reports of stress and burden among family caregivers increased due to limited face-to-face social contact [20], and a global survey showed an increase in the use of telemedicine in respite care to provide continuity of care to families and reduce caregiver burden [21].

China’s research on respite care started relatively late, and in recent years, cities such as Hangzhou, Shanghai, Beijing, and Xiamen in China have launched pilot respite care programs. Population aging has become a great challenge for many countries worldwide, and China has the largest elderly population in the world [22], and scholars have predicted that in the next 25 years, the rate of population aging in China will exceed that of Japan [23]. The State Council of China issued the “14th Five-Year Plan for National Aging Industry Development and Elderly Care Service System” in February 2022 [24], aiming to provide short-term care activities for families of the disabled elderly, to achieve the effect of improving the quality of life of the care recipients and alleviating the physical and mental stress of family caregivers, and to create an age-friendly social environment. Currently, China’s respite care services are mainly for families of the disabled elderly, related to the deepening aging society in China. Data from the National Bureau of Statistics show that by the end of 2022, there will be more than 280 million elderly people aged 60 years and above in China, accounting for 19.8% of the total national population, of which 210 million are aged 65 years and above, accounting for 14.9% of the total national population [25]. It is expected that there will be more than 77 million incapacitated elderly people in China in 2030 [26]. With the aging of the degree of aging, the proportion of home care is also gradually increasing. There is an ancient saying: “Filial piety is the foremost of all virtues”, the elderly in the home being cared for and loved is a traditional concept in China, but also one of the reasons why most of the elderly in China age at home. According to statistics, about 90% of the elderly in China are cared for by family members, and spouses and children are the main caregivers [27]. In a French study with a sample size of 4033 informal caregivers regarding support services, nearly 27% indicated the need for respite [28]. In a Chilean study on the determinants of primary caregiver burden in people with dementia, it was found that 63% of the 80 informal caregivers included presented a caregiving burden [29].

In Europe and the United States, respite care providers are primarily qualified professionals to ensure the quality of service. Currently, Chinese respite care providers are mainly social workers who lack systematic training in relevant professional knowledge and skills, and it is difficult to provide long-term services and establish stable relationships. In China, the length of schooling for most nursing graduate programs is three years, with academic master’s clinical internships requiring no less than 6 months. In contrast, professional master’s programs require no less than 18 months. Adding a Master of Geriatric Nursing Specialist to postgraduates will bring significant advantages to this field. Firstly, they have specialized knowledge and skills in geriatric nursing and are better able to understand and cope with the health problems of the disabled elderly. Secondly, with higher professionalism and a sense of responsibility, they can provide more specialized, comprehensive, and quality respite care, including life care, health monitoring, and psychological counseling. In particular, these graduate students have acquired relevant psychological education while receiving professional nursing education. In communicating with the disabled elderly and family caregivers, they can use effective psychological counseling methods to help them reduce anxiety and psychological stress. At the same time, some studies have also shown that volunteering positively impacts students’ mental health [30]. Finally, a better ability to innovate may also be available to them, providing more creative ideas and solutions for the improvement and innovation of respite care, effectively helping to reduce and alleviate the enormous pressure that aging brings to society.

Current research on respite care, with a focus on disabled elderly individuals or family caregivers, explores their willingness [31], perspectives [32, 33], needs [28, 34], and experiences [35] in using respite services. However, few studies have shown the willingness and influencing factors of professionals who volunteer to provide family respite services for disabled elderly. Therefore, this study aims to explore the willingness and influencing factors of Master of Geriatric Nursing Specialist postgraduates, a group of potential volunteers, to provide home respite care for the disabled elderly, to provide a reference to improve the quality and coverage of respite services, and at the same time, to provide a basis for the relevant governmental departments and social organizations to formulate and optimize the relevant policies and regulations.

## Methods

### Design

This study adopts Grounded Theory as a research framework and analytical methodology, a research methodology for qualitative research proposed by Anselm Strauss of Columbia University and Bar-ney Glaser of the University of Chicago in 1967, which seeks to explain and understand by generalizing concepts and theories from data to social phenomena. The grounded theory uses coding as its essential analytical tool, and we adopted Strauss and Corbin’s programmed version, which consists of open coding, axial coding, and selective coding [36]. Semi-structured interviews were conducted between June 2023 and July 2023 with Master of Geriatric Nursing Specialist postgraduates at a Hunan Province, China tertiary hospital.

### Participants

The purpose sampling method was adopted in this study to select professional master’s degree students enrolled in the geriatric nursing research direction who met the inclusion and exclusion criteria in a tertiary hospital in Hunan Province,China as research samples. A total of 12 participants were finally included in this study, represented by N1-N12. Inclusion criteria: (1) full-time professional master’s degree students of nursing in the direction of geriatrics; (2) having clear language skills, clear logical thinking, and voluntary participation in this study. Exclusion criteria: (1) Master’s degree students who have already graduated; (2) Master’s degree graduate students with equivalent education. 11 respondents were female, and one was male; the average age was 25 years old, with an age range of 23–30 years old; there were six graduate students in the first year of graduate school, four graduate students in the second year of graduate school, and two graduate students in the third year of graduate school; in terms of job title there was one nurse in charge, two nurse practitioners, and nine nurses; 3 were married, and one of them had a child; and 5 of them had had previous clinical work prior to enrolling in the program.

### Data collection

A descriptive phenomenological approach was used. In order to maintain consistency, each interview was conducted by the same researcher. Before the interviews, the researcher conducted in-depth theoretical research and received guidance from experienced experts to conduct practice interviews to improve interviewing skills. The researcher explained the concept, purpose, form, and content of respite care to the interviewee before the formal interview and determined that the interviewee had a preliminary knowledge of it to start the interview. The interview outline included: (1) How do you feel about respite care? (2) Are you willing to provide respite services to elderly people/family caregivers with such needs? Please give specific reasons. (3) What specific services are you willing to provide to elderly/family caregivers with such needs? (4) What factors motivate/hinder you to provide respite care? What factors are most important among them? How do these factors play a role? Interviews were conducted face-to-face or by voice call and lasted from half an hour to an hour each. During the interviews, leading questions were avoided, and the interviewees were encouraged to speak their minds and ask appropriate follow-up questions at the right time to obtain more prosperous and in-depth content. The recordings were immediately transcribed into text at the end of each interview and coded step by step.

### Data analysis

Based on methodological considerations and the experience of similar studies, scholars have given different recommendations on the interview sample size of grounded theory research, ranging from 5 to 35 [37]. Typically, grounded theory studies are conducted on a sample of 8 to 20 participants [38]. Based on the three-level coding approach of Strauss and Corbin’s version of grounded theory, this study first analyzed nine qualitative data. It was processed with the help of software Nvivo 12.0, which constructed a theoretical model by open coding, axial coding, and selective coding of the qualitative data, and the remaining three qualitative data are reserved for the theoretical saturation test.

#### Open coding

Open coding is a data analysis method commonly used in qualitative research, the basic idea of which is to meticulously parse the textual content of the data one by one and label or code the keywords, phrases, sentences, or paragraphs in order to analyze and understand the data in greater depth. Through continuous summarization, listing entries, and deleting contradictory initial concepts, 39 initial concepts were finally obtained to form 9 categories.

#### Axial coding

Axial coding is a theme-based method that aims to extract themes and essential concepts from data by systematically categorizing and organizing them. Often based on open coding, axial coding helps the researcher comprehend the data better and analyze or interpret the data in greater depth. The nine categories in the open coding were linked to clarify their relational connotations, resulting in 3 main categories: personal factors, service object factors, and social factors.

#### Selective coding

Selective coding is the logic of “drawing a storyline” to summarize all related conceptual categories in a theoretical model and clarify the interactions between the main categories and the core codes, and the structure of the model. The core category was established as “willingness of Master of Geriatric Nursing Specialist postgraduates to volunteer for home respite care for disabled, elderly families.” This core category was influenced by three main categories: personal, client, and social factors.

#### Theory saturation test

Strauss argues that the theory reaches a good saturation point when the additional data collected no longer leads to new categories and insights. The theoretical saturation test is the three-level coding of reserved data on this basis to test whether the coding results obtained before are accurate. In this study, the theoretical saturation test was carried out with the three pieces of reserved qualitative data, and the previous three-level coding method was repeated for a new round of coding. The results showed that the initial concept obtained in the new coding round could be obtained from the 39 initial concepts previously constructed, no new concepts and categories were found, and no new relationship structure was generated between the categories. The model reaches a saturation state in theory [39–42].

### Ethical considerations

The Ethics Committee of the Affiliated Hospital of Hunan Academy of Traditional Chinese Medicine approved the research project described here (Ref: 2023-62). All subjects provided verbal and written consent to participate, and only the researchers had access to the digital audio tapes and transcripts.

## Findings

**Fig. 1:**
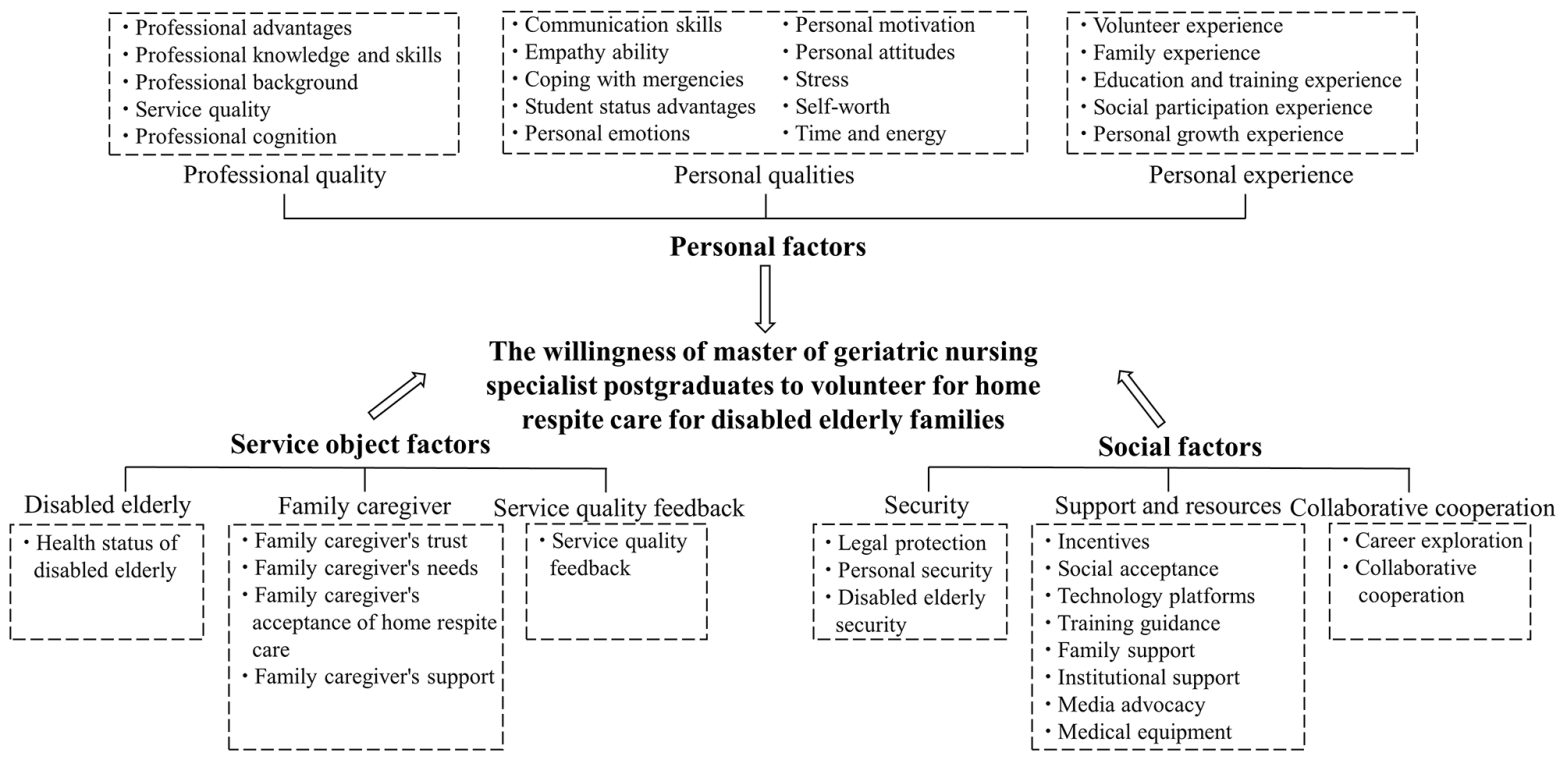
A model of influencing factors of voluntary service willingness

**Fig. 2:**
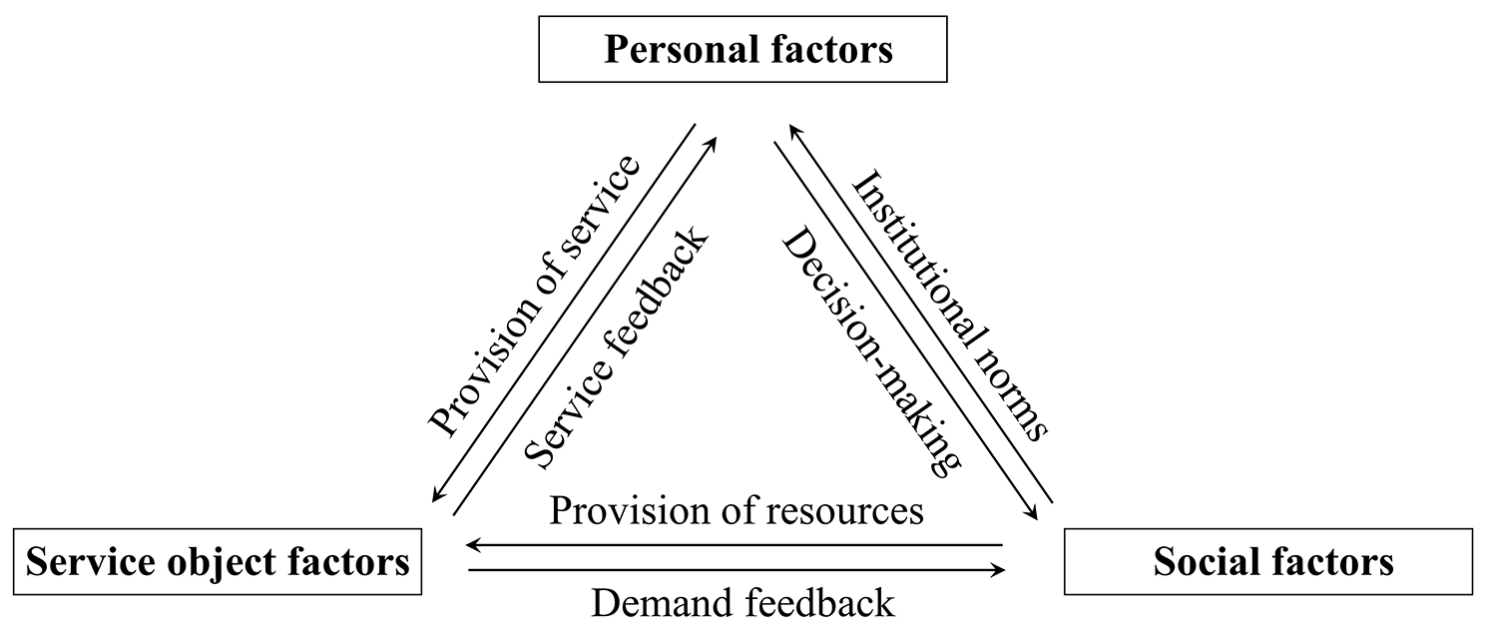
A model of the relationship among factors influencing the willingness to volunteer

In this study, through the three-level coding process of grounded theory, 39 initial concepts were refined in the open coding stage to form 9 categories of axial coding (Professional quality, Personal qualities, Personal experience, Disabled elderly, Family caregivers, Service quality feedback, Security, Support and resources, and Collaborative cooperation), with 3 main categories obtained from the selective coding (Personal factors, Service object factors, and Social factors), and based on the relationship among initial concepts, categories, main categories, and core categories, a model of influencing factors of voluntary service willingness was constructed (Fig. 1). Personal factors, service object factors, and social factors influence the willingness and behavior of graduate nursing students and are interrelated. Between personal factors and service object factors, the needs and feedback of service objects are affected through volunteers’ professionalism and personal qualities, which in turn affects the improvement of volunteers’ professional knowledge and skills and their motivation to serve; between personal factors and social factors, personal perceptions and experiential backgrounds affect individuals’ behaviors and decision-making in social environments, and at the same time, social factors also shape and affect individuals’ attitudes and behaviors and jointly affect the development and change of society; between service recipients and social factors, service recipients’ health status, social background, etc. will directly affect their needs and expectations for services, and changes and transformations in the social environment will further affect service recipients’ behaviors and needs, which will have an impact on the scale, content, and quality of the services, and promote the development of the services.A model of the relationship among personal, service object, and social factors was further constructed through further analysis of the relationship among factors influencing the willingness to volunteer (Fig. 2). The original statement after consolidation and generalization is shown below.

### Personal factors

#### Professional quality

As high-level professionals, graduate students have acquired profound professional knowledge and skills through long hours of systematic study and practical training. It not only provides them with the necessary background knowledge and operational skills but also the ability to meet the individual needs of the elderly better, thus motivating them to choose and actively participate in this volunteer work.

*As graduate students, we have deep expertise and skills that allow us to provide personalized care guidance to older adults by considering their physical condition, health needs, and personal preferences. (N5*, ***Professional advantages****)*

However, graduate students may face challenges providing home respite care for the disabled elderly. Despite their profound professional knowledge and skills, they may feel they lack sufficient experience and skills to cope with the unique needs and care requirements of the elderly. Therefore, they need to continue to learn and enhance their practical skills to adapt to the needs of this particular field. Through practice and experience, they can further consolidate their professional knowledge and skills and enhance their ability and confidence in providing home respite care services for the elderly with disabilities.

*This needs to be backed up by specialized knowledge and skills; in this regard, I still lack sufficient experience and skills. (N6*, ***Professional knowledge and skills****)*

Traditional Chinese medicine (TCM) plays a vital role in the traditional medical system, and its unique theories and techniques can provide valuable support for respite care. TCM emphasizes holistic care and conditioning, focusing on treating physical illnesses and the balance of psychological and spiritual health [43]. By incorporating the theories and techniques of TCM, the graduate students were equipped with a more holistic perspective of care. They can provide personalized nursing guidance to the disabled elderly by considering their physical condition, health requirements, and personal preferences.

*We can give full play to Chinese medicine’s therapeutic advantages and combine Chinese medicine’s theories and techniques to provide more comprehensive care services for the disabled elderly. (N7*, ***Professional background****)*

For graduate students volunteering for respite care, ensuring that seniors receive high-quality care and attention is a mission-critical and responsible task. It is not just about simply performing duties but about ensuring the quality of life and safety of the elderly. In the process, the respondents were well aware that they were still learning in the nursing field and that lack of adequate practical experience and nursing skills might adversely affect the quality of services.

*I am still in the learning process and lack sufficient practical experience and nursing skills, making it challenging to ensure the quality of my services, thus affecting the quality of life and safety of the elderly. For example, when assisting the elderly to take food and water, I need to consider their ability to swallow or other health problems to avoid accidents such as choking. In addition, when moving the elderly, I must also take special care to avoid accidents. (N1*, ***Service quality****)*

For those graduate students who have been educated in the academy for an extended period, their orientation and sense of role in their field of study often play a crucial role. Although respite care may be viewed as a primary, routine nursing task, this does not mean that graduate students will have the same attitudes about pursuing this work.N1’s perceptions highlight the potential impact of professional perceptions on individual behavioral intentions and the conflict with traditional perceptions and societal role identities.

*Because we have been studying for so many years and now have to do these essential services that some caregivers can easily handle, it doesn’t feel very comfortable for me. (N1*, ***Professional cognition****)*

#### Personal qualities

Volunteers establishing deep connections and engaging in meaningful conversations with disabled elderly individuals are the key to achieving the goals of volunteer services. They need to communicate with the disabled elderly and their families effectively, understand their needs and expectations, and be able to convey care guidance and advice clearly. However, disabled elderly individuals often face various physical and psychological challenges, challenging effective communication with them. Empathy enhances their sensitivity to the emotional needs of disabled elderly individuals, enabling them to meet their needs better and provide warm and caring caregiving services. Communication skills and empathy are interrelated, and this interrelated effect improves the quality of graduate students’ interactions with older adults and their family members, facilitating emotional support and good caregiving relationships.

*In my previous volunteer service experiences, I faced a lack of topics when communicating with the elderly. I did not know how to find suitable conversation topics and develop exciting and meaningful dialogues with them. (N4*, ***Communication skills****)*

*I think my empathy is good. I can truly understand the situation and illness of the elderly, establishing a connection with them by genuinely listening to their stories, making efforts to understand the challenges, confusion, and pain they have experienced, feeling their anxiety, loneliness, helplessness, and having a sharper insight into their needs. (N9*, ***Empathy****)*

The ability to cope with emergencies is crucial for volunteers. They need the resilience and decision-making ability to make quick and correct judgments and actions in emergencies to ensure the safety of the disabled elderly. Due to resource constraints and the complexity of the clinical practicum setting, some nursing students may not be fully exposed to various emergencies in their clinical practice and need more opportunities to respond to them. It may affect their ability to cope and self-confidence in emergencies.

*I am apprehensive that there are emergencies, and I am somewhat unsure if I can cope with them. If an older person became unwell while in my care, such as having a sudden heart attack, I’m not sure I could effectively resuscitate them. (N8*, ***Coping with emergencies****)*

Student volunteers possess sensitivity and a mindset of inclusivity in the current era. It enables them to establish emotional connections with disabled elderly individuals, through interactive communication and evoke precious memories of their loved ones and past lives. This intergenerational communication and emotional connection provide new perspectives and possibilities for elderly care, meeting the emotional needs of disabled elderly individuals and promoting mutual understanding and integration among different age groups.

*We, as students, have some unique advantages. We can accompany disabled elderly individuals, establish emotional connections with them, and evoke their memories of loved ones and the past through our communication and interaction with them. It also explains why some nursing homes have kindergartens now. (N2*, ***Student status advantages****)*

The nature of nursing work determines the deep emotional connection between healthcare professionals and patients and their families, closely related to personal empathy and caring. On the one hand, the stirring of personal emotions often ignites a strong motivation for volunteers to help and care for others, propelling them to contribute to society. On the other hand, personal motivations also play an essential role in improving one’s professional abilities, planning future career directions, caring for the well-being of loved ones, driving their participation in volunteer services, and reflecting a sense of responsibility and care for society. One interviewee (N8) expressed a different perspective from the mainstream view, emphasizing the importance of family members jointly caring for disabled elderly individuals.

*I consider myself empathetic, and I cannot bear to see the elderly struggling in pain. When I was interning at the hospital and witnessed their suffering from illness and the helplessness of their families, perhaps due to overwhelming emotions, I honestly could not control my feelings and ended up crying. (N3*, ***Personal emotions****)*

*By experiencing and understanding the current living conditions of the elderly, I can have a clearer vision and plan for my future. At the same time, it also makes me contemplate how I should provide the most suitable care and support for my parents when they grow old. (N1*, ***Personal motivation****)*

*I do not believe only graduate students can take on this role. I think undergraduate or junior college students can provide the same help and support to families with disabled elderly individuals. Moreover, I believe that families should not overly rely on this volunteer service. Family members should help and support each other, collectively taking on the responsibility of caring for the disabled elderly individuals. Volunteer services should only serve as a supplementary role and not a substitute for the responsibilities among family members. (N8*, ***Personal attitudes****)*

Stress is a common influencing factor, and nursing students may face multiple pressures, such as academic pressure, clinical environment pressure, peer pressure, and daily life pressure [44]. It should be noted that the manifestation and impact of stress factors may vary from person to person. Some graduate students may be able to actively cope with stress, continuously enhance their abilities and adaptability, and thus strengthen their willingness to engage in volunteer services. On the other hand, some graduate students may be overwhelmed by stress, leading to a decreased motivation to participate in volunteer services.

*Especially for unexpected situations, such as an emergency patient who suddenly develops a physical problem, I may feel overwhelmed and fear that the blame will be placed on me. (N4*, ***Stress****)*

*I’m worried about not being able to provide them with useful help because our purpose is to solve their problems. If we cannot help people solve their problems, it loses its original meaning. (N9*, ***Stress****)*

A sense of self-worth is one of the critical motivations for volunteers to provide respite care. By providing help and care to the disabled elderly, volunteers can gain a sense of satisfaction and fulfillment, which in turn enhances their self-worth and self-confidence. The feeling of self-worth is closely related to the individual’s perception of his or her role and significance in society, and the individual’s moral values are closely related to humanitarian sentiments.

*It will make me feel very accomplished and able to feel valued. This service is not just a duty, it is my way of giving back and dedicating myself to the community. Being able to help the elderly and their families in need makes me feel very fulfilled and satisfied, and this experience of self-worth is unparalleled. (N5*, ***Self-worth****)*

With the shortage of nurses and heavy clinical nursing workload in China [45], time and energy have become important factors influencing graduate students’ decisions. The work environment in the nursing field in China is usually characterized by high intensity and long hours, and nurses face heavy workloads every day. Nursing graduate students must cope with multiple tasks, such as internships and dissertations while undertaking their academic studies. In the face of work pressure and time constraints, it is difficult for graduate students to spare sufficient time and energy to engage in volunteer service outside of work.

*Volunteering in a location far away requires long hours of transportation and time costs. It may not be convenient for me. (N7, Time and Energy)*

*The things I face at work every day already make me feel very busy, and my time after work is already limited, and with the fatigue factor, it is tough to support me to do something outside of work. (N1*, ***Time and Energy****)*

#### Personal experience

Through personal experiences, graduate students can experience the difficulties and needs of disabled elderly individuals and develop a sense of care and respect for the elderly. Personal experiences can include volunteer experience, family experience, education and training experience, and other relevant experiences. These experiences will inspire graduate students to empathize and emotionally respond to provide respite care for families with disabled elderly individuals and lay a solid foundation for their future development in the nursing profession.

*As the league branch secretary of my undergraduate class, I had the honor to lead my classmates to volunteer in the welfare institute. Although we could only stay there for a short time, I could feel the happiness of the older adults at that moment. I do not know what will happen to them after we leave, but our presence has brought them happiness. (N2*, ***Volunteer experience****)*

*My grandmother suffered a stroke, and as a result, she is now paralyzed on one side of her body. However, her overall condition is relatively stable, and our family takes good care of her. When my grandmother first had the stroke, she felt psychologically imbalanced. Her moods fluctuated wildly, and she often needed someone to comfort her. She would cry frequently as well. Because of these experiences, when I see other elderly individuals in similar situations, I imagine that their inner emotional state might also be problematic. I deeply understand that they need someone to comfort and support them. I believe that, like my grandmother, disabled elderly individuals need our care and companionship to help them through this challenging period. (N3*, ***Family experience****)*

*I remember when I obtained a certificate as a professional infant caregiver during my undergraduate studies, and I deeply felt the importance of providing care and support to others. This experience made me realize that both infants and elderly individuals need our care and companionship. Additionally, after giving birth to my child, I experienced the assistance of a lactation consultant who provided home visits. This experience made me understand the importance of professional nursing and support in addressing issues. I deeply understand the challenges and needs of families with disabled elderly individuals, and I recognize the need for professional care and assistance. I realize that I can and am willing to provide such services, and I see the demand for them in the market. (N5*, ***Education and training experience****)*

*I have participated in community service activities, and the elderly are grateful that we can help them focus on their health. I think health education and focusing on the lifestyles of the elderly can be effective in preventing some diseases, so I think it is necessary to provide home respite services. (N7*, ***Social participation experience****)*

*I am a member of the Communist Party and have served in the army. Thanks to the cultivation of the Party and the army, I deeply understand the importance of serving the public. I am convinced we should provide more care and services to the disabled elderly to assist them in their old age. (N9*, ***Personal growth experience****)*

### Service object factors

#### Disabled elderly

Stable health conditions in the disabled elderly increase graduate students’ confidence and competence, whereas unstable or complex health conditions may trigger feelings of worry and challenge in graduate students. In order to increase graduate students’ willingness to participate, it is necessary to provide relevant training and support to enhance their ability to cope with various health conditions.

*If the health condition of disabled elderly individuals is stable, I may feel more confident in providing respite services because I know their condition is relatively manageable. However, if the health condition of disabled elderly individuals is unstable or complex, I may worry that I cannot handle it. In such cases, I may choose not to participate or seek other suitable ways to assist the disabled elderly individuals. (N1*, ***Health status of disabled elderly****)*

#### Family caregiver

Family caregivers’ trust in volunteers is an important factor in determining their willingness to serve, as trust is the basis for a cooperative relationship. Secondly, the needs of family caregivers are key to volunteers’ willingness to serve, for example, understanding their needs for time, energy, and emotional support and volunteers’ ability to provide appropriate support. Meanwhile, family caregivers’ acceptance of respite care directly affects whether volunteers can participate in the service successfully. Finally, the support of family caregivers is a crucial factor in facilitating volunteers’ participation. Their support can provide volunteers with motivation, encouragement, and recognition and enhance their willingness and motivation to participate. Therefore, building trust, understanding family caregiver needs, enhancing acceptance of respite care, and obtaining family caregiver support are vital elements motivating graduate students to provide respite care.

*I am concerned that family caregivers may not trust me because I lack experience caring for disabled elderly individuals. However, I understand their worries as they have deep emotions and a sense of responsibility towards the disabled elderly. They may worry that outsiders may not understand and meet the particular needs of the elderly or that outsiders may hurt them. However, I believe that through building trust and communication, we can work together to provide the best care and support for the disabled elderly. (N6*, ***Family caregiver’s trust****)*

*One primary concern is that I worry that the needs of the family members of the disabled elderly may exceed my capabilities, and I may not be able to provide the high-quality service they expect. Therefore, I will carefully assess my abilities and experience to determine my competence for this job. (N5*, ***Family caregivers’ needs****)*

*Some family caregivers may feel that asking us to care for the disabled elderly may make others think they are not filial. However, in reality, our service is to assist them rather than to replace their responsibilities. In our culture, respecting the elderly and filial piety are essential values. I understand the concerns of family caregivers, but at the same time, I hope they can face their decisions openly, understand their abilities and limitations in caring for the disabled elderly, and actively seek professional assistance if needed. (N1*, ***Family caregiver’s acceptance of home respite care****)*

*Caring for the disabled elderly is challenging, and although we will do our best to take care of them, we are not omnipotent. There may be mistakes or imperfections in some matters. I hope that in such situations, family caregivers can understand us, work with us to find solutions, and provide us with support and encouragement, rather than blame and criticism. (N11*, ***Family caregiver’s support****)*

#### Service quality feedback

Service quality feedback refers to the evaluation and feelings of family members towards the respite care provided by previous volunteers, directly affecting their expectations and attitudes toward the current services. Positive service quality feedback can build trust and satisfaction, thereby increasing family members’ acceptance of new volunteers. On the other hand, negative service quality feedback may lead to distrust and suspicion from family members, reducing their acceptance of volunteer services and potentially having a negative impact on the entire respite care plan for the disabled elderly.

*If the previous volunteers left a bad impression or if they feel that we are just going through the motions and cannot truly solve their problems, it will be difficult for us to carry out our work. (N9*, ***Service quality feedback****)*

### Social factors

#### Security

Graduate students’ concerns about security are rooted in the uncertainty surrounding accidents and dispute resolution. The safety risks faced by volunteers during the service provision not only affect their interests and safety but also relate to the well-being and protection of the disabled elderly. Legal protection can provide volunteers with clear responsibilities and obligations, enabling them to provide services more confidently without excessive worry about legal risks. Establishing relevant legal policies will provide better legal support and guidance for volunteers while ensuring more reliable service protection for the disabled elderly.

*If an elderly person has an accident, such as suddenly fainting, falling, developing a sudden illness, or other situations that are out of our control, compensation and dispute resolution need to be considered, as well as avoiding unnecessary disputes and controversies. (N5, Disabled elderly security*, ***Personal safety****)*

*It would be best if there were relevant legal policies to clarify the responsibilities and obligations of volunteers because this can provide better legal support and guidance for volunteers, and more reliable service protection for the elderly. (N10*, ***Legal protection****)*

#### Support and resources

Incentives provide visible and tangible rewards, meeting individuals’ self-actualization needs, while social recognition provides intangible but more enduring motivation, making volunteering a respected and praised social behavior. Currently, there is still room for improvement in society’s understanding and recognition of volunteer service. Many people have not deeply understood the value and significance of volunteering, and the work of volunteers has not received enough attention and acknowledgment.

*It is necessary to provide some incentives, such as issuing volunteer certificates, giving extra points when selecting scholarships, or providing a small stipend. (N8*, ***Incentives****)*

*Through various channels such as promotional activities, media coverage, or social media platforms, it is important to communicate the stories and contributions of volunteers to the public, increasing social recognition for volunteers. (N12*, ***Social acceptance****)*

With the continuous development of technology, technological platforms play a significant role in volunteer work. By utilizing the internet and mobile technology, volunteers can communicate and coordinate with families of disabled elderly individuals more conveniently. Technological platforms can provide online information exchange and resource-sharing functionality, enabling volunteers to schedule respite care more effectively and improve work efficiency. Therefore, exploring and applying appropriate technological platforms is necessary to increase volunteers’ willingness to participate. In addition, training and guidance are crucial factors that influence volunteers’ participation in respite care for disabled elderly individuals. Volunteers must undergo professional training and acquire the necessary knowledge and skills before providing services. The training should cover elderly care, communication skills, and crisis management. Real-life case studies should guide it to enhance volunteers’ professional competence and service quality. Regular guidance and feedback should also be provided to help volunteers continuously grow and improve throughout their service.

*The ideal scenario would involve a professional technological platform that facilitates mutual selection. On the platform, disabled elderly individuals and their families can post their needs and invite volunteers while also being able to view basic information and the service experience of volunteers. Similarly, volunteers can post their skills and service information on the platform while understanding the needs and family situations of disabled elderly individuals. The technological platform should also adequately consider the needs of family caregivers and provide mechanisms for their feedback and evaluation. (N5*, ***Technology platforms****)*

*While our professional backgrounds may be related to elderly care, the challenges and complexities in practice may exceed our expectations and require prior training. (N4*, ***Training guidance****)*

Family support, whether economic, emotional, or time-related, directly affects the willingness and ability of family members to participate in volunteer services. Family support can alleviate their pressure during service and enhance their motivation to continue participating. Institutional support can also enhance volunteers’ professional competence, service quality, and enthusiasm for participation. Necessary training, resources, and management support can ensure that volunteers are competent and can provide high-quality services.

*Family support is also important to me. When I am feeling conflicted internally, even if I go to do the service, I will not feel happy. My family’s understanding, care, and support can motivate me more. (N1*, ***Family support****)*

*If we could receive support from hospitals and schools, the entire activity would be more organized and sustainable. For example, they can assist in providing venues, promoting the event, and even providing necessary materials and equipment. It not only makes volunteers feel cared for and supported but also encourages more people to participate in this act of kindness. (N3*, ***Institutional support****)*

Media publicity can expand the influence and awareness of volunteer services and enhance social acceptance. When volunteers receive widespread coverage and praise, they feel that their work is recognized by society, forming a virtuous cycle. However, media publicity also has some limitations. Issues such as excessive emphasis on adverse events, incomplete reporting, and one-sided information may raise doubts and misunderstandings among the public about elderly care services, thereby affecting the willingness of student volunteers. Therefore, it is necessary to strengthen the supervision and guidance of media publicity, ensuring the authenticity and comprehensiveness of information to establish public trust in elderly care services.

*Media publicity is indeed crucial. Many people are unfamiliar with this service, so we need to do more publicity to make more members of society aware of its existence and value. We can organize public welfare activities or collaborate with celebrities to attract more attention and participation. (N4*, ***Media advocacy****)*

Currently, there is a gap in the availability of medical equipment at home compared to hospitals, which may limit the availability of volunteers to provide quality care in the home environment. For the disabled elderly with diverse needs and complex health problems, more specialized and advanced medical equipment is required to support their care. Inadequate equipment and facilities may result in volunteers feeling powerless in performing their work, affecting the range of care measures they can implement and the support they can provide.

*Compared to hospitals, medical equipment at home visits is less comprehensive and complete than there, which may make me feel powerless in delivering my services. (N3*, ***Medical Equipment****)*

#### Collaborative cooperation

The industry that provides respite care for disabled elderly individuals has not received sufficient attention and development. For individuals aspiring to engage in social work, this service may become one of their career choices. Respite care in our country is still in its early stages and faces many challenges and issues, such as establishing service standards, qualification recognition mechanisms, and personnel training. Addressing these problems and further promoting professional development and closer collaboration will bring broader prospects for development.

*This type of service can form a profession similar to being a part-time worker. Some people are interested in this type of social work, even if it only provides a small subsidy of a few dozen yuan per hour. (N2*, ***Career exploration****)*

*The involvement of doctors is also crucial. Their professional knowledge and guidance can improve the quality of service and ensure that the elderly receive timely and accurate medical support. (N6*, ***Collaborative cooperation****)*

## Discussion

Based on the grounded theory, this study conducted semi-structured interviews with 12 Master of Geriatric Nursing Specialist postgraduates, summarized and extracted relevant information from the interview data through three-level coding. Finally, it established a model of influencing factors of voluntary service willingness and a model of the relationship among factors influencing the willingness to volunteer. These models deeply reflect the essential elements that affect the volunteers of Master of Geriatric Nursing Specialist postgraduates to provide home respite care for disabled elderly families under the background of Chinese traditional culture, education and medical environment.

The research of Lily Dongxia Xiao et al. [46] shows that Chinese nursing students have a higher attitude and preference toward caring for the elderly than Australian nursing students, which may be related to the two different cultural backgrounds of individualism and collectivist cultures. The results of a study on volunteer service of a sample of Indian college students show that there are two primary motivations for volunteer service: poor- and upper-class students volunteered to secure educational opportunities. In contrast, middle-class students volunteered to secure employment [47]. Different from this result, volunteer service in China is mainly influenced by traditional social concepts and family education. The research results of Yao, Ching-Teng et al., Taiwan, China [48] show that essential predictors of medical students’ willingness to take care of the elderly include their feelings of getting along with the elderly, their grandparents being the main caregivers in childhood, and their experience of providing volunteer services for the elderly. The results of this study are similar to those of this study. Both express medical students’ pursuit of social responsibility and concern for vulnerable groups.

In one particular volunteer care study, only 58% of volunteers were willing to provide respite care after training [49]. Similar to some of the findings in this study, a qualitative study in Chile found that potential volunteers providing respite care to bedridden elderly people also wanted training in medical procedures to provide meaningful care to the elderly, and wanted a feedback process to maintain continuity of service [50]. In one Ireland study, respite care providers specifically underlined that while they recognize unmet support needs, because of under-resourcing and underprovision, they cannot always assume responsibility for such needs [51]. This also reflects the need to find more ways to increase the importance of respite care providers when respite care resources are insufficient. A survey in the United Kingdom found that although person-centred care is widely recognized, it is not embedded in the organizational cultures of all local providers of respite care [52].

In Chinese culture, respect for the elderly is an important traditional value emphasizing respect, care, and concern for the elderly [53]. This study shows that although Master of Geriatric Nursing Specialist postgraduates in China, influenced by the traditional cultural background, show high motivation in volunteering to provide home respite care to families of the disabled elderly, the many pressures and challenges brought by China’s complex healthcare environment and education system make it more difficult for them to make this decision. Their enthusiasm for volunteering can be further stimulated through proactive reform measures and policy support, thereby enhancing the quality and coverage of home respite care to meet the needs of the growing elderly population. We, therefore, put forward the following strategies and policy recommendations.

### Strategies for improving respite care willingness

Establishing a support system is an integral part of enhancing the willingness to provide respite care, which includes setting up relevant support institutions or teams in schools and community organizations to provide graduate students with the necessary guidance, training, and resource support. For example, collaborating with relevant organizations,such as municipalities, nongovernmental organizations, charities, and religious institutions, to help the health system establish respite care facilities and services [54], Providing a practical place for graduate students.For example, the College of Nursing at Auburn University has cooperated with the REACH Community Respite Ministry [55]. Through regular activities such as seminars and graduate student forums, exchanges and cooperation among graduate students can also be promoted to enhance their awareness and enthusiasm for volunteering.

With the aging of the population and the complexity of care issues for the elderly, interdisciplinary collaboration is particularly prominent in geriatrics [56], and it is a recognized and well-developed care coordination model [57]. Home respite care involves multiple disciplinary fields, such as sociology and psychology. Therefore, the establishment of an interdisciplinary teamwork mechanism to work together on family assessment, social support, psychological counseling, and health monitoring, which enables experts and graduate students from various fields to work together on research and practice related to respite care, helps to increase their comprehensive knowledge of and interest in the field and integrates the resources and expertise of all parties to provide a more holistic and integrated service.The realization of interdisciplinary cooperation may face disciplinary barriers, different role cognition, and different working methods. Therefore, it is necessary to strengthen communication and exchange between various disciplinary fields, establish mutual trust and respect cooperative relations, clarify their respective roles and responsibilities, explore suitable working methods and methods, and coordinate interests and goals.

Due to the unpaid nature of volunteering, incentive policy support is crucial to enhance the willingness of graduate students to volunteer. Chen Chen et al. [58] argued that a combination of spiritual incentives and material rewards can better enhance the motivation of volunteers. Government departments can introduce relevant incentive policies to encourage graduate students to participate in home respite care for families of the disabled elderly and give them certain rewards or honors, as well as provide volunteers with the necessary legal protection and appropriate financial support to improve the attractiveness of graduate student volunteerism.

To summarize, we can make efforts in three aspects: establishing a support system, an interdisciplinary cooperation mechanism, and incentivizing policy support. The implementation of these strategies will help to cultivate the interest and participation of more graduate students and provide better home care for the disabled elderly and promote the development of respite care in China.

### Reflections and suggestions on the development of respite care

Improving our country’s respite care service system is the foundation for promoting the development of respite care. Currently, respite care services in China have yet to be fully utilized, and the service scope is relatively narrow [31]. The government should increase support for respite care, but it is difficult to rely on local governments’ financial support alone. It should expand the funding sources for services, encourage social enterprises and charitable organizations to play their social responsibilities and support respite care. At the same time, respite care agreements can be signed with elderly care institutions or community health services to extend professional services to families. It is worth noting that for informal respite care, China currently needs a unified management department, resulting in service quality can not be effectively guaranteed. Therefore, the future should establish relevant management departments and a robust regulatory and evaluation mechanism for respite care, strengthening the supervision and evaluation of service quality and effect. At the same time, it should also enhance the construction of respite care personnel and constantly revise and improve the laws and regulations related to respite care to ensure sustainable development and improvement.

Seeking diversified service models is vital in enhancing the adaptability and flexibility of respite care [59]. Currently, China’s main respite care modes are home respite care and institutional respite care, which have yet to be further divided. China can draw lessons from the successful experience of foreign respite care and combine it with the actual situation in China to carry out detailed division to meet the needs of different groups. However, due to significant variations in the needs of different groups, remote medical care models could be considered complementary respite care models [60]. Additionally, the government should encourage community volunteer organizations and social groups to participate in respite care by providing companion, social, and recreational services. It’s also essential to encourage innovative service models to meet the needs of different groups.

Establishing an information-sharing platform is an important means to improve the accessibility and transparency of respite care. Currently, information about respite care is scattered, incomplete and needs more flexibility. Families need more knowledge of existing services and help to access relevant information and support [61]. The government should establish an information-sharing platform for respite care to centrally collect and organize relevant information, including service providers, service content, and fee information. It can also provide online booking and consultation services to facilitate access to respite care information and support for those in need. For the necessary technical and resource support, the government can invest human and financial resources to establish advanced information technology systems. For the possible conflicts of interest and cooperation problems among various departments and institutions in the information-sharing process, it is necessary to establish a cooperation mechanism for information sharing and clarify the responsibilities and obligations of all parties. For possible data security and privacy protection issues, it is necessary to formulate relevant data protection laws, regulations, and technical measures to ensure that personal information is not leaked or abused.

In summary, measures such as improving the respite care system, establishing diversified service models, and creating an information-sharing platform will enhance service quality and coverage, promote respite care development, and improve the quality of life for disabled elderly individuals and their families. The government, social organizations, and community residents should work together to form a collective effort to further advance respite care development.

### Limitations

This study has several limitations. First, the study’s sample size is a relatively small sample. However, we follow the theoretical saturation principle of grounded theory and believe that the sample size is sufficient to meet the study objectives. Second, the study data was collected at a tertiary hospital in Changsha, China, which may limit the generalizability of the proposed influencing factor model, especially in other countries with different cultural backgrounds. However, the range of basic information of the participants in this study, such as age, grade, job title, work experience, and marital status, is considered an advantage in the study because these participants can better represent the views of this group. Therefore, we believe the data referenced in this study can represent the traditional Chinese cultural background and the views of master of geriatric nursing specialist postgraduates in China’s current educational and medical environment.

## Conclusion

This study aims to explore in-depth the willingness and influencing factors of Master of Geriatric Nursing Specialist postgraduates to volunteer for home respite care for disabled, elderly families. Through qualitative interviews, three main categories of influencing factors were identified: individual, Service object, and social factors. It is recommended to enhance the training and guidance of respite care providers and family caregivers, promote the implementation of collaborative care plans, and focus on improving social factors, including developing comprehensive policies and providing more social support and recognition. We hope this study can provide valuable references and insights for improving the quality of respite care services for disabled elderly individuals and promoting the development of the geriatric nursing profession. Additionally, Future research can involve participants from different geographical regions and cultural backgrounds, consider incorporating quantitative research methods to validate further and quantify the identified influencing factors, establish a more comprehensive and accurate model, and also consider focusing on the long-term effects of volunteer participation to assess respite care’s sustainability better.

## Data Availability

The dataset generated for this study will be made available from the corresponding author on a reasonable request.
